# The Evaluation of Determinants and Impacts of Co-Production in Healthcare: A Research Protocol for OPAT in Cystic Fibrosis

**DOI:** 10.5334/ijic.5568

**Published:** 2021-04-30

**Authors:** Marta Marsilio, Andrea Gramegna, Floriana Fusco, Eleonora Gheduzzi, Giovanna Pizzamiglio, Francesco Blasi, Chiara Guglielmetti

**Affiliations:** 1 Department of Economics, Management and Quantitative Methods (DEMM), Università degli Studi di Milano, via Conservatorio, 7, 20122, Milan, Italy; 2 Department of Pathophysiology and Transplantation, Università degli Studi Di Milano, IT; 3 Fondazione Irccs Ca’ Granda Ospedale Maggiore Policlinico, Via Francesco Sforza, 35, 20122, Milan, Italy; 4 School of Management, Politecnico di Milano, Via Lambruschini 4, 20156 Milan, Italy

**Keywords:** co-production, evaluation, impact, determinants, health, OPAT, cystic fibrosis

## Abstract

**Introduction:**

Co-production is more and more considered as a promising tool for dealing with the main challenges in the health sector (e.g., growing rates of chronic diseases, budget constraints, higher patients’ expectations of the quality and the value of services, equity to access of care, etc.). However, there is still little evidence on co-production determinants and impacts.

**Description:**

This research protocol aims to present a framework to assess the determinants and impacts of the co-productive approach in healthcare delivery on patients, professionals, and providers from economic, organisational, and clinical perspectives. To this end, the paper examines the co-produced outpatient parenteral antimicrobial therapy (OPAT), applied to cystic fibrosis patients in an Italian hospital. A mixed methods approach will be adopted and data will be collected through semi-structured interviews and surveys of patients, caregivers, and professionals; biological samples of patients; archival sources. Then, the analyses to be performed are the following: (i) cost evaluation, (ii) content, (iii) descriptive and inferential statistical, (iv) microbiome analysis, and (v) desk analysis.

**Conclusion:**

The research protocol contributes to both theoretical and practical knowledge. It represents the first attempt to develop a systematic analytical framework for the evaluation of co-production in healthcare. Moreover, the findings gathered within the study will provide evidence to support policy makers and managers in decision-making and managerial processes within the health service.

## Introduction

### Background and rationale of the study

Co-production is seen as a promising tool for dealing with challenges in the health sector [1,2,3]. The rates of chronic diseases are growing, as well as the expectations of patients regarding the quality of services [4]. This puts the healthcare systems under pressure to contain costs without detracting from the high quality of care. To this end, policy makers (e.g. [5]) have promoted the development of a more personalised care based on new relational models, in which patients, their informal carers, and local communities share responsibilities with care providers, thus enabling them to feel part of the team and more willing to continue self-care after discharge. In the last months, this need has appeared even more crucial due to the COVID-19 outbreak and the consequent risks [6,7,8].

The concept of service co-production has its roots in the late 1970s and early 1980s (e.g. [9,10]). In 1996, Ostrom and her colleagues defined it as:

“the processes through which inputs, used to provide a good or a service, are contributed by individuals who are not in the same organization” [11, p. 1073].

At that point, the attention to co-production had declined until the arrival of the global financial crisis (i.e. 2008 and 2009), when pluralistic models of governance and the sense of community found favour as a concept again [12]. Theoretical and empirical contributions on the topic have increased significantly in the past decade, and co-production has been investigated in different disciplines and applied in numerous policy sectors [2,13]. Nonetheless, there is still a lack of consensus on many aspects, such as: the actors involved (only service users or any external organisations), the phases of the process (many or few steps of the service cycle: co-commissioning, co-design, co-delivery, and co-assessment), the nature of the involvement (only voluntary or also involuntary) [13,14]. The consequence is that the concept lacks a unique definition [12].

In public service literature, co-production is broadly understood as the active involvement of lay actors (individual users, groups of users, or communities) partnering with professionals in any phase of the public service cycle (e.g. [12,15,16]) or just during the implementation phase (e.g. [13,17]).

In service management literature, co-production is assumed to be one of the most important elements of value co-creation, and, specifically, it concerns the customer participation, involvement, and engagement in the production (i.e. co-design or shared delivery) of goods and services [18,19,20].

Despite this vagueness of boundaries, scholars agree in considering healthcare to be one of the most theoretical and empirically promising application fields for co-production [2,20]. Batalden [21, p. 2] describes co-production of health as:

“the interdependent work of users and professionals who are creating, designing, producing, delivering, assessing, and evaluating the relationships and actions that contribute to the health of individuals and populations”.

Thus, according to this definition and the broad understanding of the concept in public and service management literature, health co-production is considered as a wide variety of activities in which health providers (public or private) and health consumers (patients and/or carers or communities) voluntarily work together to produce some benefits in any phase of the service cycle (i.e. commissioning, design, delivery, and assessment). In this variety of activities, health co-production includes for instance, the shared work of clinicians and health consumers working together to improve healthcare processes and systems (e.g. [22,23]) such as: the co-delivery of learning/training activities by professionals and experts by experience (e.g. [24,25]) and patients’ councils in health organisations collaborating in strategic decisions (e.g. [26,27]). Our preference for the term ‘health provider’ in the definition rather than ‘professionals’ allows us to go beyond the dyadic dimension (i.e. the ‘doctor–patient relationship’) and to move into a system dimension able to consider the managerial and organisational implications of co-production [28,29].

Health co-production can be affected by the healthcare general context, that includes variables belonging to the environment which can create opportunities or constrains, affecting how the co-produced initiative unfolds [30]. Moreover, the type of co-produced service (e.g. acute vs chronic care) and the service delivery channel (e.g. community-based primary care vs hospital/specialist care) can influence the variation in propensity to co-produce of health care provider and health consumers [20]. If the process has been largely standardized by the service provider and the exchanges between providers and patients are limited in terms of number and time (e.g. acute care in hospital setting), the patient has limited opportunities to provide useful resources for improving the existing understanding of the illness and treatment procedures. Instead, if a service required an extended involvement of patients during time and the effectiveness of treatment can benefit from their experience and knowledge about the disease (e.g. ongoing and chronic illnesses), the provider and patient would be more willing and able to co-produce, exerting a greater impact on care process’s results [31]. In this direction, a recent bibliometric analysis on co-production studies in health [32] highlights that the elderly is the most investigated target, as it is the most critical segment of the population in terms of chronicity and comorbidity and with greater health and social care needs.

There is an increasing interest in the potential benefits of co-production on the healthcare system. So far, research reports positive impacts on *providers*, in terms of services’ effectiveness, efficiency, innovativeness, and personalisation [26,33,34]; on *users*, in terms of increasing wellbeing, quality of life, satisfaction, self-esteem, self-confidence, and empowerment (e.g. [23,24,34]); and on *clinicians*, in terms of learning, increased empathy, better relationships with patients and job satisfaction (e.g. [24,33,35]). However, co-production is not a panacea [36]. The collaboration among actors may be partially or totally unsuccessful, producing negative or inconsistent results on the users, providers, and professionals involved (e.g. [33,37,38]). The literature has also highlighted that a successful co-production could be affected by manifold lay actors’ and providers’ determinants, such as patients’ and clinicians’ skills, competences, and personal motivations or the organisational ability to manage the co-produced activity (e.g. [3,35,39,40]).

Despite the growing number of publications, there is a need to further investigate the effects of co-production in healthcare [20,32,41]. First, the large majority of existing studies are single exploratory or narrative case or qualitative studies where the impacts are assessed only using in-depth or semi-structured interviews, with limitations in terms of internal and external validity [42] and in terms of risk of common method bias when data are gathered from a single stakeholder. Moreover, many studies have adopted self-reported questionnaires, but very few with validated scales and large samples [43,44]. These methodological choices make the results hardly generalisable. Second, the majority of studies have assumed a mono-dimensional and a mono-stakeholder approach and lack a comprehensive evaluation of the co-production. Third, despite the fact that co-production in the co-design phase or in learning/training activities has been widely investigated, very few studies have been carried out on the co-delivery of care [32].

With the aim of filling these gaps, this paper presents a research protocol aimed at evaluating the determinants and the overall impacts of a co-produced service, by analysing a co-delivered chronic care treatment (i.e. outpatient parenteral antimicrobial therapy, hereafter OPAT) in the primary Adult Cystic Fibrosis (CF) Centre of a large academic hospital in Italy. To this end, a generalisable evaluation framework has been developed, including dimensions usually neglected in existing literature (i.e. managerial and organisational).

### Co-production in OPAT treatment

OPAT is a potentially useful method for delivering intravenous antimicrobials in the outpatient setting [45]. First adopted in the USA in the mid-1970s, the need for OPAT has been progressively recognized in Canada, UK, Australia, New Zealand, and European countries. Currently, OPAT is used to treat a wide range of infections, including soft tissue infections, osteomyelitis, endocarditis, and pulmonary exacerbations of cystic fibrosis (CF). CF has proven to be one the most suitable settings where OPAT can be applied and studied [46]. CF is a genetic disease, dominated by pulmonary symptoms and the establishment of chronic pulmonary infections with bacteria [46]. A large increase in the adult CF population is expected in the next decade [47]. To date, the therapeutic goal in CF is to slow down the progression of the disease, in order to safeguard the patients’ survival into adulthood. According to Burgel and colleagues, western European countries’ forecasts indicate that the 50% increase in the overall number of CF patients by 2025 corresponds to an increase by 75% in the adult population [47]. Acute exacerbations of chronic respiratory infection are fought through an antibiotic treatment, that can be delivered by three routes: oral, inhaled aerosol, or intravenous. In the presence of severe exacerbations, intravenous therapy can be required on average for 10–14 days but, especially in the more advanced stages of the disease, longer times may be necessary [48]. Hence, the option of OPAT offers CF patients the opportunity to undergo the needed antibiotic treatment at home, with less of an impact on daily life in comparison to the inpatient option. There are several service models for administering OPAT, but typically long-term patients carry out at least a portion of needed therapy at home, after hospital discharge and having received specific training [49]. OPAT requires multiple steps of coordination and integration of care between patients/caregivers and several professionals/healthcare providers [50]. During the home treatment period, home care assistance, general practitioners and/or hospitals collaborate with patients at different levels. They can monitor patients’ care progress, provide medications, draw blood, and provide catheter care. In this regard, existing literature stresses the importance of delivering OPAT programmes through coordinated and multidisciplinary professionals and organisations [51,52].

Thus, from a managerial point of view, OPAT can be considered an example of co-produced treatment that specifically involves users in the co-delivery of the service [53]. Patients are asked to participate actively and act as co-producers of their care, assuming the tasks of delivering the antibiotic treatment at home, recognising and promptly communicating adverse reactions, and contributing to effective interactions with clinicians at various levels [45]. Co-production in chronic patients, such as CF adult patients, involves learning some skills and self-management strategies (see Schulman et al. (2013) [54]), but it goes far beyond self-management. OPAT is not simply a standard daily routine, but requires a transformation of the service delivery model, in order to ensure on the one hand, the integration of care, and on the other, the active participation of patients/co-producers and their caregivers.

Evidence claims that OPAT, when compared to inpatient treatment, allows a decrease of hospital admission costs and length of stay, and turns out to be safe and clinically effective, offering also benefits in terms of reduced hospital-acquired infections [45,55,56]. Some qualitative studies found a generally high satisfaction of patients’ with OPAT. At the same time, they raised some critical issues on safety and the need to improve patient-centeredness in OPAT care, taking into account more the patients’ personal and material resources to effectively support self-management at home [49,57].

The enthusiasm for OPAT must then be cautioned; it is a complex practice to manage both from a clinical and organisational point of view. Antibiotic administration carries some risks; it is potentially toxic. Successful co-production of OPAT requires frequent and attentive involvement from healthcare providers deeply and at multiple levels (multidisciplinary health professionals, different care settings, etc.) and requires a strong patient committed effort in the co-production of the treatment. Consequently, health providers and staff are dealing with two important managerial challenges: (i) setting and running the delivery processes, coordinating the interdependency between both organisational, professional, and patient’s tasks; and (ii) getting the patient to be engaged in the healthcare plan [53].

### Research aims

The main research question is ‘what are the determinants and the overall impacts of the OPAT co-produced treatment for CF patients in comparison to traditional treatment?’ To answer this research question, the study that will be performed will address two main objectives:

#### Aim 1

The evaluation of the determinants and the impacts/outcomes of co-produced OPAT treatment compared to those of traditional inpatient treatment will be the primary aim of the study.

**Figure 1:**
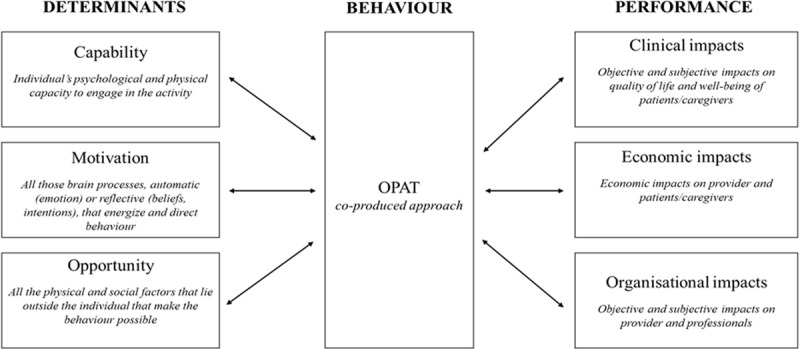
The study framework - an adapted version of COM-B Model.

The COM-B model framework will be adopted for the analysis. This model proposes that the change of behaviour is affected by capability, opportunity, and motivation [58]. It has been effectively used in health research to categorise the determinants of co-production (e.g. [40,59]). In this study, the model will be adjusted (i) adding the performance according to the three main relevant dimensions which emerged from literature on co-production (clinical, economic, and organisational) and (ii) considering the three main stakeholders involved in a co-produced health service (hospital/providers, professionals, and patients and their caregivers) (***Figure 1***).

The study will also aim to evaluate the longitudinal impacts/outcomes of a new OPAT procedure, which will be introduced shortly in the investigated hospital and which is intended to formalise eligibility criteria and the pathway for the treatment of CF patients in order to increase the accessibility to this promising treatment. The same operationalisation described in Aim 1 will be used.

## Methodology

### Study design

The research is a single case study. Given the two aims, the study will be designed according to two research approaches:

- *Aim 1*: A cross-sectional study will be performed to evaluate OPAT therapy in comparison with inpatient antibiotic intravenous therapy.
- *Aim 2*: A longitudinal study will be adopted to assess any variations that occur from T0, namely the introduction of the new procedure, to T1, after 12 months.

### Study setting and OPAT model of care

The study will be conducted at the Respiratory Unit and Adult Cystic Fibrosis Centre Fondazione IRCCS Ca’ Granda Ospedale Maggiore Policlinico (subsequently referred to as ‘the Centre’ or ‘CF Centre’). This is a regional reference centre for CF in Lombardy and holds a high number of clinical records compared to the national average. The Centre cares for approximately 320 patients with CF and is home to a wide array of experimental research and clinical trials. It started to adopt the OPAT approach in 1995. The CF Centre is staffed with a multidisciplinary team that includes five physicians, three nurses, three physiotherapists, two dieticians, one social worker, and one psychologist. Patients will be invited to participate in the study during their routine clinical journey, hospitalisation, and in day-hospital or outpatient settings. The staff will be involved during working hours, in a way that is compatible with their working requirements.

**Figure 2:**
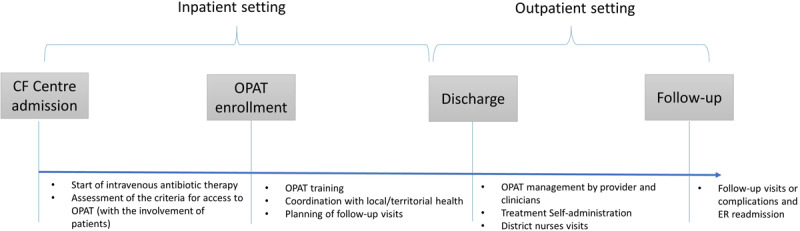
Depiction of OPAT flow in the CF Centre.

Specifically, the co-production process in OPAT at the CF Centre always starts during hospitalisation, then it can continue at home if patients meet a number of predefined criteria (which involve clinical and non-clinical personal and social aspects of the patient’s life, and the presence of adequate local health units). These criteria, as well as the patient’s willingness to carry out therapy at home, are also assessed through a productive dialogue and information exchange between a multidisciplinary physicians’ team and patients. If patients are enrolled, during the hospitalisation, specialist nurses carry out training sessions in which patients and caregivers are provided with the knowledge and skills needed to self-deliver an intravenous antimicrobial in the outpatient setting and to identify/manage any adverse reactions. Vascular access devices are inserted by specialists of another hospital unit (PICC-TEAM). Before and after the discharge, the coordination and the integration between CF Centre and local health is essential. Before the discharge, the CF centre (i) contacts the local health unit in order to supply patients with the devices needed and drugs for the therapy; (ii) activates home care assistance that guarantees a visit by a district nurse every day and informs the general practitioner of the patient’s situation; (iii) schedules with the patient the date for a periodic follow-up at the CF Centre or in a local walk-in centre. The OPAT process may be interrupted due to adverse drug reactions or malfunctions of intravenous access, and patients may be forced to be hospitalised (***Figure 2***).

### Target population

**Figure 3:**
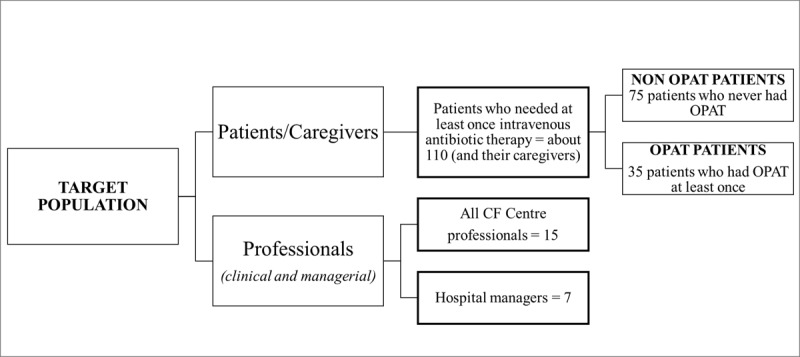
The study target population.

The target population will include adult patients who need the intravenous antibiotic therapy at least once, and their caregivers and staff, both clinical and managerial (***Figure 3***). In consideration of the critical issues of the transition process from paediatric care (where OPAT is a parents-directed care) to adult care (where OPAT can be considered a co-produced treatment), we have decided to exclude patients under transition process from paediatric to the adult unit or who have been actively followed up in the adult unit for less than two years. Moreover, we exclude patients who never required intravenous antibiotic therapy or refuse to participate in the study.

The expected patients sample size is estimated according to 2018 data and amounts to 110 patients hospitalised for intravenous therapy. Approximately 35 adults will be assigned to OPAT and 75 to inpatient intravenous therapy. One caregiver per patient will be invited to participate. We will ask to the patient to indicate at least one informal (family member, friends) or formal caregiver who provides the stable assistance during OPAT.

Patients eligible for inclusion in the study will receive a copy of the patient information sheet, which will provide details on the purpose of the study and the type of clinical information collected. Once patients have had the opportunity to consider and understand the significance of their participation, they will be asked to provide their written informed consent. The participation will be voluntary, refusal to participate will involve no penalty or loss of benefits, and the subject may discontinue participation at any time without penalty or loss of benefits to which the subject is otherwise entitled. The study will be performed in accordance with the Helsinki Declaration, the version adopted by the 18th World Medical Assembly in June 1964 and its subsequent amendments, as well as the International GCP and applicable regulatory requirements.

The study will include all the CF Centre clinician staff and the hospital managerial staff (i.e. the Chief Executive Officer, the Director of Clinical Practice, the Chief Administrative Officer, the Chief Information Officer, the Chief Operating Officer, the Chief Nursing Officer, and the Director of Medicine). Inclusion and exclusion criteria are summarised in ***Table 1***.

**Table 1:** Inclusion and exclusion criteria for the participants.


SAMPLE	INCLUSION CRITERIA	EXCLUSION CRITERIA

Patients *(and their caregivers)*	needed at least once intravenous antibiotic therapy; in follow up at the adult unit for at least two years; agree to participate in the study, by signing informed consent.	never required intravenous antibiotic therapy; under transition process from paediatric to the adult unit or has been actively followed up in the adult unit for less than two years; refuse to participate in the study.

Professionals *(clinicians and hospital manager)*	agree to participate in the study	refuse to participate in the study


### The variables of the framework

According to co-production literature, the specific context of cystic fibrosis, and the research aims, the adapted COM-B model has been operationalised as follows:

**Determinants** include patients/caregivers, staff, providers, and system factors that can foster or hinder OPAT adoption. Specifically:

- *Capability* considers the individual’s psychological and physical capacity to engage in the activity. This category includes, for example, patients’ and professionals’ skills, competences, knowledge, education, and resource (time, money, etc. availability) (e.g. [40,60,30]);
- *Motivation* concerns all those brain processes, automatic (emotion) or reflective (beliefs, intentions), that energise and direct behaviour. It includes, among others, the individual and psychological attitudes and values, the level of patients’ self-efficacy, and trust [3,17,30];
- *Opportunity* refers to all the physical and social factors that lie outside the individual that make the behaviour possible. It includes, for instance, institutional structures and processes, such as formal rules, allocation of responsibilities, training, design of service delivery channels, and space [40,30].

**Performance** concerns the impacts of OPAT on patients/caregivers, staff, and providers, from a:

- *Clinical perspective*, considering the impacts on patients through objective clinical indicators (i.e. safety, effectiveness of care, adverse events, quality of life, and patient-reported outcomes, and acquisition of new respiratory bacteria), and subjective indicators (i.e. quality of life, patient activation/engagement, quality of care perception, and customer satisfaction). The study also aims to evaluate any change in: (i) microbiology in terms of traditional microbiology and microbiome analysis (indicators of bio-diversity of the lower airway microbiota) on sputum samples; (ii) indicators of local inflammatory activity between the two groups measured by median IL-8 and Neutrophil Elastase level on sputum samples.
- *Economic perspective*, intending to measure both direct and indirect costs for provider and patients/caregivers.
- *Organisational perspective*, including the impacts on staff, measured by objective indicators (e.g. turnover rate, absences, injuries, and work-related ill health, near miss, and medical errors) and subjective indicators (burnout, satisfaction, healthcare professional engagement, OPAT-related risk perception, perceived support, and change involvement etc.) and on the healthcare provider (e.g. impact on patient flow logistics, and collaborations and knowledge management tools).

The specific measures will be identified through literature review. The appropriateness of questions to the target population and the comprehensibility of the instructions will be tested with a preliminary pilot study with a small sample of eight patients and three staff members.

### Data collection and analysis

The study will adopt a mixed methods approach [61], that will increase the breadth and range of study findings, capturing relevant information that might be missed by relying on only one research method and which will in general, enhance and strengthen the research results [62,63].

Specifically, data will be collected through (a) interviews with patients, caregivers, and clinical/managerial staff; (b) a survey administered to patients, caregivers, and clinical/managerial staff; (c) biological samples of patients (sputum); and (d) archival sources. Then, the following qualitative and quantitative analyses will be performed:

- *Content analysis*. The semi-structured interviews will be audio-taped and then transcribed verbatim. Content analysis will be performed through a qualitative data management software package (NVivo 12, QSR International, Daresbury, UK). The interviews will be aimed at collecting the perceptions and experiences of patients, caregivers, and staff about OPAT (Aim 1) and the new OPAT procedure (Aim 2). They represent a useful tool to help the interpretation of quantitative data and to obtain further interesting insights.
- *Descriptive and inferential statistics*. Quantitative data will be statistically analysed. A descriptive statistic will be carried out to describe the sample characteristics. As the study aims imply, in order to detect differences between groups of different sizes, propensity scores will be calculated and used both as a controlling variable in the multivariable model and to construct matched cohorts. To compare cross-sectional data scores, differences in continuous data will be analysed using t-tests or Wilcoxon signed rank tests, as appropriate. All statistical analyses will be conducted using SPSS and R.
- *Cost evaluation analysis*. This will be performed from the provider and patient/caregiver perspectives estimating the differential costs existing between hospital therapy and home therapy. The aim is to verify if there is a cost saving or, on the contrary, an increase compared to inpatient therapy, both for the hospital (provider) and the patients/caregivers. From the provider perspective, medical costs (e.g. drugs, devices, and costs for ED visits/hospitalisation), administrative costs (e.g. non-clinical services related to hospitalizations, overhead, etc.), personnel costs will be considered. The specific single items of the differential cost analysis will be identified comparing the inpatient and outpatient co-produced pathways, interviewing the CF Centre’s staff and also taking into account the new standard procedure. Then, data will be retrieved from the provider’s archival sources. From the patient/caregiver perspective, non-medical costs, which include missed work and travel expenses, will be included. This data will be collected during the patients’ and caregivers’ interviews.
- *Microbiome analysis*. Microbiome will be evaluated on sputum, and colour, pH and standard microbiology will be assessed on fresh sputum. Cytokine analysis will be performed on soluble sputum using Cytokine array I for the Randox Evidence Investigator, and MPO, MMP9, neutrophil elastase, and desmosine presence will be evaluated using an ELISA assay.
- *Desk analysis*. Desk analysis will be performed on archival and secondary administrative data collected (e.g. SDO and personnel data), provided by the hospital, in order to obtain objective clinical and organisational impact indicators.

Specifically, to accomplish Aim 1 – *The evaluation of the determinants and the impacts/outcomes of co-produced OPAT treatment compared to those of the traditional inpatient treatment*, a cross-sectional study comparing OPAT with inpatient therapy will be developed using content analysis and descriptive and inferential statistics to evaluate the determinants (capability, motivation and opportunity); while, content analysis, descriptive and inferential statistics, cost evaluation analysis, microbiome analysis and desk analysis on archival data to evaluate the performance. To address Aim 2 – *The evaluation whether and how new procedure impacts on the dimensions and stakeholders of the model* a longitudinal study comparing OPAT before and after new procedure will be performed, using content analysis and descriptive and inferential statistics referring to the determinants, while content analysis, descriptive and inferential statistics, cost evaluation analysis and desk analysis on archival data to evaluate the performance.

***Table 2*** summarises objectives, variables, and methods of analysis.

**Table 2:** Aims, variables, and methods matrix.


AIMS	STUDY DESIGN	VARIABLES (COM-B MODEL)	METHODS

The evaluation of the determinants and the impacts/outcomes of co-produced OPAT treatment compared to those of the traditional inpatient treatment	Cross-sectional study, comparing OPAT with inpatient therapy	– Determinants *(Capability; Motivation; Opportunity)*	Content analysis of semi-structured interviews of patients, caregivers, and professionals; descriptive and inferential statistics on questionnaires administered to patients, caregivers and professionals;

		– Performance *(Clinical; Economic; Organisational)*	Content analysis of semi-structured interview of patients, caregivers, and professionals; descriptive and inferential statistics on questionnaires administered to patients, caregivers, and professionals; cost evaluation analysis; microbiome analysis; desk analysis on archival data

2. The evaluation whether and how new procedure impacts on the dimensions and stakeholders of the model	Longitudinal study, comparing OPAT before and after new procedure	– Determinants *(Capability; Motivation; Opportunity)*	Content analysis on semi-structured interview to OPAT patients, caregivers, and professionals; descriptive and inferential statistics on questionnaires administered to OPAT patients and professionals;

		– Performance *(Clinical; Economic; Organisational)*	Content analysis on semi-structured interview to OPAT patients and professionals; descriptive and inferential statistics on questionnaires administered to OPAT patients and professionals; cost evaluation analysis; desk analysis on archival data.


## Discussion

The study will aim to analyse and evaluate a co-production practice (home antibiotic therapy— OPAT) in an Italian primary centre for cystic fibrosis.

Findings are expected to show co-production is beneficial for both provider and patients/caregivers, in terms of a perceived increase of patients’ quality of life and well-being, patient satisfaction, efficiency (cost-savings), and effectiveness of care. Moreover, some challenges will be expected to be found in organisational dimensions for staff and provider.

The research is innovative at different levels. At a conceptual level, (i) OPAT is interpreted as a co-produced process involving healthcare providers and consumers. To the best of our knowledge, this issue appears as a novelty; (ii) the study develops a comprehensive framework for the overall assessment of co-production in health. The evaluation framework assumes a multi-dimensional (clinical, economic, organisational) and multi-stakeholder (provider, staff, patients/caregivers) approach, overcoming literature limitations on the analysis of impacts of co-production in health service delivery mainly focussed on specific stakeholders (e.g. impacts on patients) or dimensions (e.g. economic) or phases other than the co-delivery of care (e.g. learning/training).

At methodological level, the case study goes beyond widely used narrative single perspective qualitative research, since it proposes several multi-stakeholder’s data collection methods (interviews and surveys to patients, staff, caregivers; biological samples and archival sources) that will allow rival explanation, triangulation and logic model, thus strengthening the validity and generalizability of the study [42]. Furthermore, the study combines qualitative evidence, with quantitative descriptive and inferential statistics on questionnaires administered to patients, caregivers, and professionals.

At an empirical level, looking at the literature on OPAT, the study presents some elements of originality:

- Clinical domain: This is the first study to evaluate the use of microbiome in the OPAT setting along with usual clinical indicators. Data about a possible protective effect of OPAT on the acquisition of new respiratory bacteria are currently lacking. In this regard, the microbiome analysis based on the analysis of the 16S ribosomal RNA gene is a recent microbiological technique enabling both the broad detection of bacteria in a sample and their phylogenetic identities. The investigation aims to answer the question of whether the outpatient setting has an impact on respiratory microbiome with positive effects on lung health. The study will give new insights on the role of outpatient versus inpatient management of CF patients;
- Economic domain: this is the first study which verifies OPAT economic cost-effectiveness in an Italian setting;
- Organisational domain: the study analyses the possible impact of OPAT on the workload and welfare of healthcare professionals; this has been almost completely neglected by previous literature.

Accordingly, the findings can offer new insights both on the role of co-production in the health sector and on the OPAT management of CF patients. Moreover, the evaluation model could be applied to other co-produced health practice.

Despite these contributions, the study presents some limitations concerning the chosen design and sample size. First, the data will be drawn from a single centre. Moreover, although the centre is one of the largest in Italy and despite broad inclusion criteria, the target population (patients and staff) is quite limited. So, the generalisability of the findings should take place with some prudence. For this reason, future research directions will have to replicate the model in different centres and clinical contexts (e.g. other pathologies already using OPAT).

## Conclusion

The study combines a typically managerial perspective (co-production) with a clinical perspective (OPAT therapy). It is expected to make a useful contribution both in academic and practical knowledge. It represents the first attempt to develop and empirically adopt a systematic analytical framework for the overall evaluation of the co-production of healthcare. Moreover, it will provide policy makers and healthcare managers with a practical assessment tool for supporting decision-making processes and the management of the co-delivery of healthcare service with patients, caregivers, and other stakeholders of the healthcare service network, by improving the understanding of impacts of co-produced practices.
